# Relationship between CT-derived cervical muscle mass and quality, systemic inflammation, and survival in symptomatic patients undergoing carotid endarterectomy

**DOI:** 10.1093/bjsopen/zrae114

**Published:** 2024-10-23

**Authors:** Nicholas A Bradley, Karamonique Dosanj, Sharon Yen Ming Chan, Alasdair Wilson, Tamim Siddiqui, Rachel Forsythe, Campbell S D Roxburgh, Donlad C McMillan, Graeme J K Guthrie

**Affiliations:** Academic Unit of Surgery, University of Glasgow, Glasgow, UK; Department of Vascular Surgery, NHS Tayside, Dundee, UK; Department of Vascular Surgery, NHS Lothian, Edinburgh, UK; Department of Vascular Surgery, NHS Grampian, Aberdeen, UK; Department of Vascular Surgery, NHS Lanarkshire, East Kilbride, UK; Department of Vascular Surgery, NHS Lothian, Edinburgh, UK; Academic Unit of Surgery, University of Glasgow, Glasgow, UK; Academic Unit of Surgery, University of Glasgow, Glasgow, UK; Academic Unit of Surgery, University of Glasgow, Glasgow, UK; Department of Vascular Surgery, NHS Tayside, Dundee, UK

## Abstract

**Background:**

Sarcopenia appears to be associated with inferior outcomes in surgical conditions. Chronic systemic inflammation confers an inferior long-term prognosis in cardiovascular disease and is associated with the development of sarcopenia. The aim of this study was to describe the prognostic role of sarcopenia assessed using computed tomography (CT)-derived body composition analysis and systemic inflammation in patients undergoing carotid endarterectomy for symptomatic carotid stenosis.

**Methods:**

In this retrospective cohort study, patients undergoing carotid endarterectomy for symptomatic carotid stenosis between 1 January 2011 and 1 October 2021 at four referral centres were included. The C3 skeletal muscle index and C3 skeletal muscle density were recorded from preoperative CT images. Systemic inflammation was assessed using the preoperative systemic inflammatory grade (SIG). The primary outcome was overall mortality during the study interval.

**Results:**

A total of 618 patients were included, with a median follow-up of 69 (interquartile range 34–85) months. On univariable analysis, age greater than or equal to 75 years (*P* < 0.001), American Society of Anesthesiologists (ASA) grade greater than II (*P* < 0.001), low C3 skeletal muscle index (*P* = 0.002), low C3 skeletal muscle density (*P* < 0.001), SIG greater than or equal to 2 (*P* < 0.001), and low L3 derived skeletal muscle index (*P* < 0.001) were associated with increased mortality, whereas body mass index greater than or equal to 25 kg/m^2^ was associated with decreased mortality (*P* = 0.023). On multivariable analysis, age 75 years or older (HR 2.17 (95% c.i. 1.58 to 2.97), *P* < 0.001), ASA grade greater than II (HR 2.06 (95% c.i. 1.35 to 3.12), *P* < 0.001), low C3 skeletal muscle density (HR 1.84 (95% c.i. 1.33 to 2.54), *P* < 0.001), and SIG greater than or equal to 2 (HR 1.63 (95% c.i. 1.33 to 1.99), *P* < 0.001) were independently associated with increased mortality.

**Conclusion:**

Cervical CT-derived muscle mass and density, and markers of systemic inflammation, such as systemic inflammatory grade, may be associated with an inferior long-term prognosis after carotid endarterectomy.

## Teaser

Sarcopenia and systemic inflammation appear to influence prognosis in a range of disease states, including cardiovascular disease. This retrospective multicentre study included 618 patients undergoing carotid endarterectomy for symptomatic lesions. Low cervical muscle density and increased magnitude of systemic inflammation were independently associated with inferior long-term survival.

## Introduction

Carotid endarterectomy largely remains the treatment of choice for symptomatic carotid lesions after transient ischaemic attack (TIA) or cerebrovascular attack (CVA) and continues to make up a significant proportion of the UK vascular surgery caseload^1,2^. The majority of operations in the UK are performed for symptomatic disease, with asymptomatic disease less aggressively treated (which is in contrast to worldwide practice)^2^. Furthermore, the mainstay of UK practice is surgical endarterectomy rather than stent placement^2^. The prophylactic intent of surgery, which aims to reduce the risk of future stroke, highlights the importance of patient selection for surgery *versus* best medical therapy. Disease-specific factors, such as degree of stenosis and interval from event to operation, are commonly used in clinical practice to determine the potential risk and benefit in individual patients^1^.

Sarcopenia is a chronic condition characterized by progressive loss of skeletal muscle mass and function^3^. Patients with sarcopenia are typically frail, with inferior physiological reserves, and appear to be at risk of inferior outcomes in major vascular and non-vascular surgery^4,5^. Sarcopenia can be quantified using computed tomography (CT)-derived body composition (BC) analysis (CT-BC), allowing measurement of cross-sectional muscle and fat areas and density^6^. Low muscle mass (skeletal muscle index (SMI)) and quality (skeletal muscle density (SMD)) appear to be associated with inferior outcomes in patients undergoing endovascular repair of abdominal aortic aneurysms^7,8^.

Activation of the systemic inflammatory response (SIR) is recognized in a range of chronic illnesses, including atherosclerotic disease^9^, to be an important aetiological and prognostic factor associated with the development of sarcopenia^3^. The SIR can be measured using prognostic scoring systems, such as the neutrophil:lymphocyte ratio (NLR), the modified Glasgow prognostic score (mGPS), and the composite systemic inflammatory grade (SIG), each of which has been shown to yield prognostic value in a range of disease states^10–12^. Loss of muscle mass and systemic inflammation appear to offer independent prognostic value in patients with and without cancer^13,14^. There is a paucity of data documenting these relationships in patients with cardiovascular disease, particularly cerebrovascular disease.

Historically, CT-BC has been most commonly performed on abdominal imaging, typically at the L3 vertebral level. More recently, cervical muscle mass and quality at the C3 vertebral level have been quantified via CT and appear to influence prognosis in patients with head and neck cancer^15^. The association between sarcopenia and outcomes has been explored in patients undergoing carotid endarterectomy; however, to date, C3 skeletal muscle area (SMA) has not been reported, with masseter area the most commonly reported measure to date^16,17^. Similarly, the prognostic value of the SIR in carotid surgery has been explored, with several authors reporting inferior outcomes in patients with an elevated NLR^18,19^. Alternative measures of the SIR, and the interaction between muscle mass and inflammation, remain unreported in this patient cohort.

The present study examines the association between CT-derived cervical muscle mass and quality, systemic inflammation, and survival in patients undergoing carotid endarterectomy.

## Methods

### Patient selection

Patients undergoing carotid endarterectomy were retrospectively identified from pre-existing theatre records at four tertiary referral centres in Scotland, UK, representing cases drawn from four health boards (NHS Grampian, NHS Lanarkshire, NHS Tayside, and NHS Lothian). Specific procedural technique and choice of post-procedure secondary prevention strategy were at the discretion of each institution, though practice was broadly similar between sites throughout the study interval. To perform CT-BC, a preoperative planning CT angiogram at the C3 level was required; the choice of whether this was required or whether case planning was based on duplex ultrasonography was at the discretion of the operative team. Consecutive cases undergoing carotid endarterectomy between 1 January 2011 and 1 October 2021 were screened for inclusion. Patients with active malignancy, active infection (both due to the potential confounding effect on the variables of interest), and missing or corrupted (for example due to artefacts from dental implants) CT images or SIG were excluded. West of Scotland Research Ethics Committee approval was obtained for this study (Reference 21/WS/0146; approval granted 23 November 2021).

### Baseline data collection

Clinical, demographic, and co-morbidity data were recorded from electronic case records and patients’ community health records. Co-morbidity was assessed using American Society of Anesthesiologists (ASA) grade, which was recorded from operative records and subgrouped (less than or equal to II and greater than II) in keeping with previous literature^20^. Age (less than 75 and greater than or equal to 75 years) and body mass index (BMI) (less than 25 and greater than or equal to 25 kg/m^2^) were considered as categorical variables. Patients with missing baseline data were selectively excluded from relevant analyses.

### Outcomes of interest

The primary outcome was overall mortality during the follow-up interval. The secondary outcome was 5-year survival, chosen as this reflected a clinically relevant outcome in this patient group. Outcome data were obtained from the Community Health Index (CHI) registry, a routinely available registry maintained at a national health board level and populated from both primary and secondary care data. Specific cause of death was not available from this registry.

### CT-derived body composition analysis

Body composition analysis was performed using arterial-phase CT images acquired as part of surgical planning at the C3 vertebral level. Using methodology previously described^21^, sternocleidomastoid and paravertebral muscle groups were manually measured using the freeware program ImageJ (National Institutes of Health, Bethesda, MD, USA; version 1.53)^22^, using muscle tissue thresholds of −29 to +150 Hounsfield Units (HU). This generated the C3 SMA, which was normalized to height^2^ to generate the C3 SMI. The C3 SMD was also recorded, which, as per established practice, was not normalized^6^. Additionally, using the formula proposed by Swartz *et al*.^23^, the L3 derived skeletal muscle index (dSMI) was calculated from the C3 SMA. To determine optimal thresholds of continuous variables for dichotomization of body composition parameters into ‘normal’ and ‘low’, the ‘surv_cutpoint’ function of the ‘survminer’ R package was used, using the maximally selected rank statistic technique. In keeping with prior reports^24^, preliminary analyses showed a difference in median C3 SMI and L3 dSMI between sex (*P* < 0.001) and BMI (*P* < 0.001) subgroups, and in median C3 SMD between sex (*P* < 0.05) subgroups. Therefore, sex-specific (C3 SMI, L3 dSMI, and C3 SMD) and BMI-specific (C3 SMI and L3 dSMI) thresholds were derived to account for these differences, in keeping with established methodology for abdominal CT in patients with cancer^24^. Outcomes were compared between subgroups of CT-BC parameters.

### Inflammatory profiling

Institutional policy during the study interval was to admit patients to hospital on the evening before surgery, where preoperative blood work was routinely performed as part of existing patient care. NLR (from absolute neutrophil and lymphocyte counts) and mGPS (from C-reactive protein and albumin) were calculated based on preoperative blood investigations using previously described methodology^10,11^ (*Table S1*). NLR and mGPS were then combined into SIG, as previously reported^25^, and outcomes compared between groups of SIG 0 (considered ‘non-inflamed’) *versus* SIG 1 (considered ‘mildly inflamed’) *versus* SIG greater than or equal to 2 (considered ‘inflamed’).

### Statistical analysis

Differences between continuous variables were assessed using the Kruskal–Wallis and Mann–Whitney tests and differences between categorical variables were assessed using the chi-squared test. The relationship between covariates and survival was assessed using a Cox proportional hazards model; covariates were initially interrogated in univariable analysis and those with univariate *P* < 0.05 were included in a multivariable model. To avoid confounding due to multicollinearity, C3 SMI and L3 dSMI were modelled in separate multivariable models, with the values for C3 SMI rather than L3 dSMI preferentially reported due to cervical CT-BC being the main covariates of interest. Time-to-event analyses were performed using the Kaplan–Meier method, with differences between cohorts assessed using the log rank test. Where time to event survival data did not reach a median survival, the mean (95% c.i.) values are reported. Percentage 5-year survival and percentage s.e. were calculated using censored survival data for each CT-BC parameter and SIG subgroup, and absolute differences between subgroups were compared. *P* < 0.05 was considered statistically significant. To account for potential selection bias, patients excluded due to missing CT or SIG data were compared with the final study cohort. Analyses were performed using SPSS^®^ (IBM, Armonk, NY, USA; version 28.0) and RStudio 2022.02.01.

## Results

There were 970 patients screened for eligibility; after exclusions based on missing CT or SIG data, 618 patients were included in the final analysis. The median follow-up was 69 (34–85) months. There were 178 deaths during the follow-up interval; the mean survival in the entire cohort was 101.4 (95% c.i. 96.3 to 106.5) months and 5-year survival was 77.0% (s.e. 2.0%).

A total of 188 (30.4%) patients were aged 75 years or older, 436 (70.8%) were male, 426 (69.4%) had a BMI greater than or equal to 25 kg/m^2^, 397 (68.2%) had ASA grade greater than II, and 306 (49.8%) and 312 (50.2%) cases were performed for CVA and TIA respectively (*Table 1*). Thresholds of cervical CT-BC parameters associated with an inferior prognosis are shown in *Table 2*. There were 328 (53.4%) patients with low C3 SMI, 270 (44.2%) patients with low L3 dSMI, and 189 (30.7%) patients with low C3 SMD. There were 325 (52.8%) patients with SIG 0, 197 (32.0%) patients with SIG 1, and 94 (15.3%) patients with SIG greater than or equal to 2. The association between cervical CT-BC parameters and baseline clinical characteristics is shown in *Table 3*.

**Table 1:** Summary baseline demographic and clinicopathological characteristics of the study population of patients undergoing carotid endarterectomy for symptomatic carotid stenosis (*n* = 618)

**Age (years)**	
<75	430 (69.9)
≥75	188 (30.4)
Median (i.q.r.)	70 (63–75)
**Sex**	
Male	436 (70.8)
Female	182 (29.2)
**BMI (kg/m^2^)**	
<25.0	187 (30.6)
≥25.0	426 (69.4)
Median (i.q.r.)	27.1 (24.4–30.3)
**ASA grade**	
≤II	182 (31.8)
>II	397 (68.2)
**Diagnosis**	
CVA	306 (49.8)
TIA	312 (50.2)
**Thirty-day CVA**	
Yes	10 (1.6)
No	608 (98.4)

Values are *n* (%) unless otherwise indicated. i.q.r., interquartile range; BMI, body mass index; ASA, American Society of Anesthesiologists; CVA, cerebrovascular attack; TIA, transient ischaemic attack.

**Table 2:** Threshold values of cervical CT-derived body composition analysis parameters associated with inferior survival in patients undergoing carotid endarterectomy for symptomatic carotid stenosis (*n* = 618)

**Low C3 SMI**	
Male, BMI <25.0 kg/m^2^	≤14.8 cm^2^/m^2^
Male, BMI ≥25.0 kg/m^2^	≤17.7 cm^2^/m^2^
Female, BMI <25.0 kg/m^2^	≤12.8 cm^2^/m^2^
Female, BMI ≥25.0 kg/m^2^	≤11.8 cm^2^/m^2^
**Low C3 SMD**	
Male	≤43.7 HU
Female	≤37.4 HU
**Low L3 dSMI**	
Male, BMI <25.0 kg/m^2^	≤50.0 cm^2^/m^2^
Male, BMI ≥25.0 kg/m^2^	≤50.5 cm^2^/m^2^
Female, BMI <25.0 kg/m^2^	≤32.7 cm^2^/m^2^
Female, BMI ≥25.0 kg/m^2^	≤37.1 cm^2^/m^2^

CT, computed tomography; SMI, skeletal muscle index; BMI, body mass index; SMD, skeletal muscle density; HU, Hounsfield Units; dSMI, derived skeletal muscle index.

**Table 3:** Association between baseline clinical characteristics and systemic inflammation, subgrouped by CT-derived body composition analysis parameters, in patients undergoing carotid endarterectomy for symptomatic carotid stenosis (*n* = 618)

	Normal C3 SMI	Low C3 SMI	*P*	Normal C3 SMD	Low C3 SMD	*P*
Age ≥75 years	76 (26.7)	111 (33.8)	0.055	117 (27.3)	71 (37.6)	0.010*
Female sex	127 (44.6)	53 (16.2)	<0.001*	154 (35.9)	26 (13.8)	<0.001*
BMI ≥25 kg/m^2^	201 (70.5)	225 (68.6)	0.605	286 (67.1)	140 (74.9)	0.056
ASA grade >II	180 (67.7)	217 (69.6)	0.627	262 (65.2)	135 (75.4)	0.014*
CVA at diagnosis	142 (50.2)	160 (49.7)	0.905	208 (49.1)	98 (52.7)	0.409
SIG ≥2	31 (10.9)	63 (19.2)	0.015*	62 (14.5)	33 (17.5)	0.089

Values are *n* (%). Patients with missing BMI (required for sub-grouping based on threshold of CT-BC parameter) were selectively excluded from relevant analyses. *P* values generated through linear-by-linear chi-squared analyses. *Statistically significant. CT, computed tomography; SMI, skeletal muscle index; SMD, skeletal muscle density; BMI, body mass index; ASA, American Society of Anesthesiologists; CVA, cerebrovascular attack; SIG, systemic inflammatory grade.

**Fig. 1:**
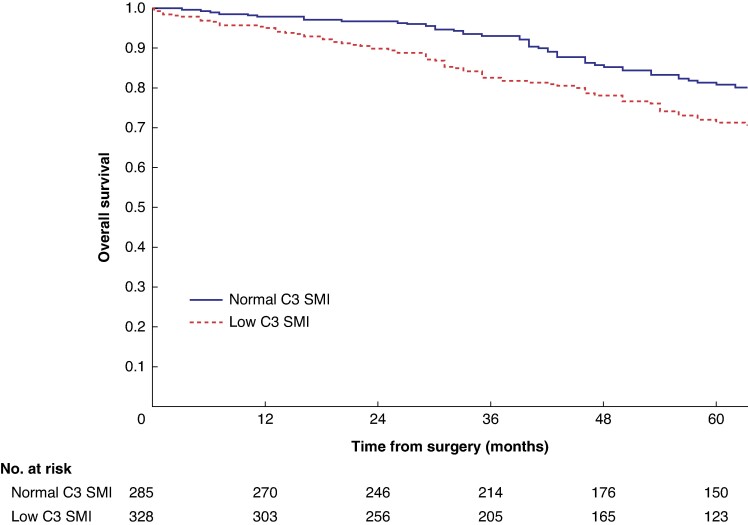
Overall survival in patients undergoing carotid endarterectomy for symptomatic carotid stenosis when subgrouped by C3 skeletal muscle index (*P* = 0.002) SMI, skeletal muscle index.

**Fig. 2:**
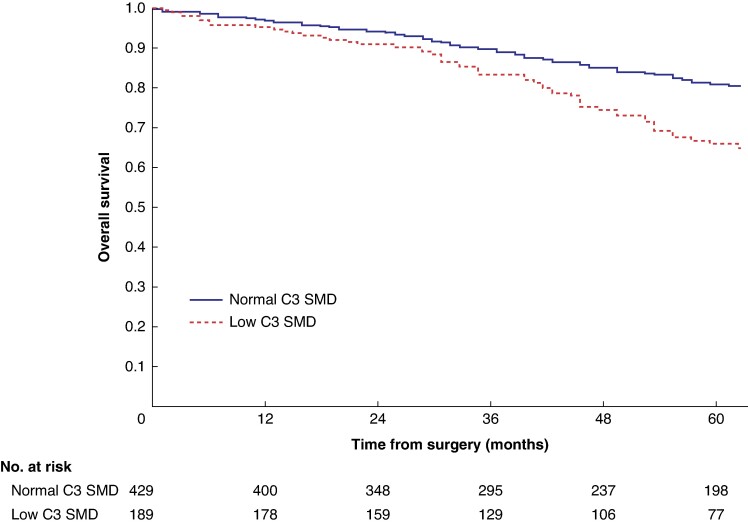
Overall survival in patients undergoing carotid endarterectomy for symptomatic carotid stenosis when subgrouped by C3 skeletal muscle density (*P* < 0.001) SMD, skeletal muscle density.

**Fig. 3:**
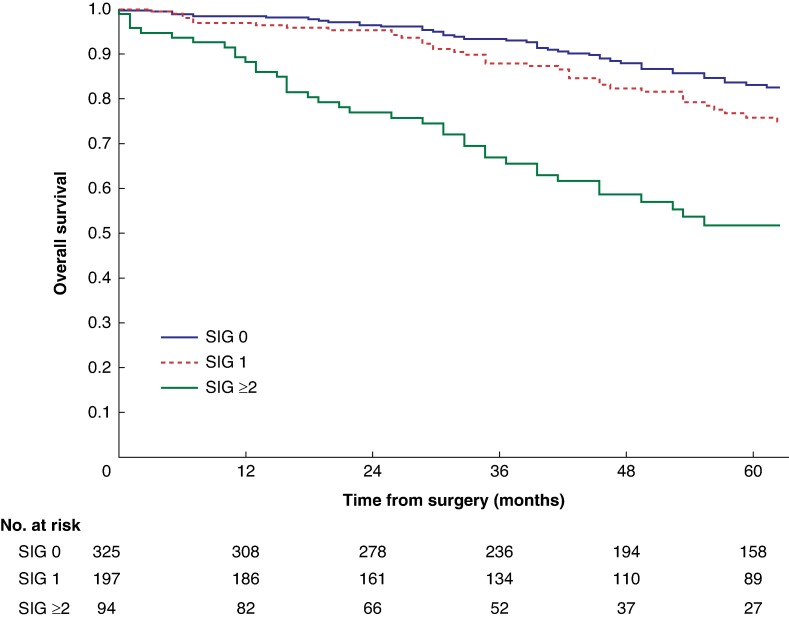
Overall survival in patients undergoing carotid endarterectomy for symptomatic carotid stenosis when subgrouped by systemic inflammatory grade (*P* < 0.001) SIG, systemic inflammatory grade.

**Fig. 4:**
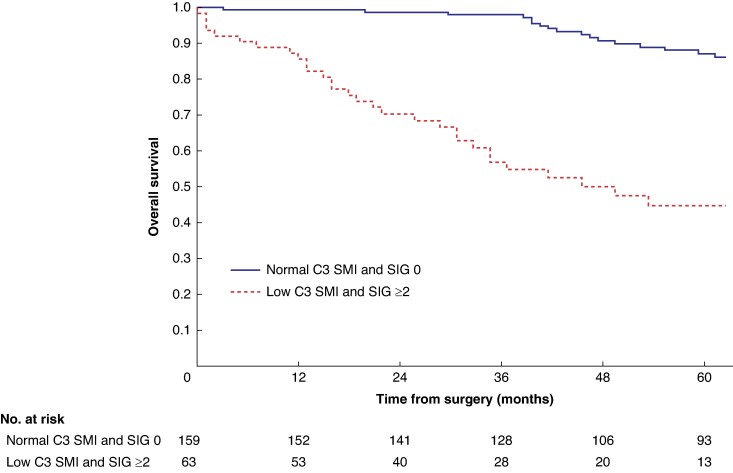
Overall survival in patients undergoing carotid endarterectomy for symptomatic carotid stenosis when subgrouped by C3 skeletal muscle index and systemic inflammatory grade (*P* < 0.001) SMI, skeletal muscle index; SIG, systemic inflammatory grade.

**Fig. 5:**
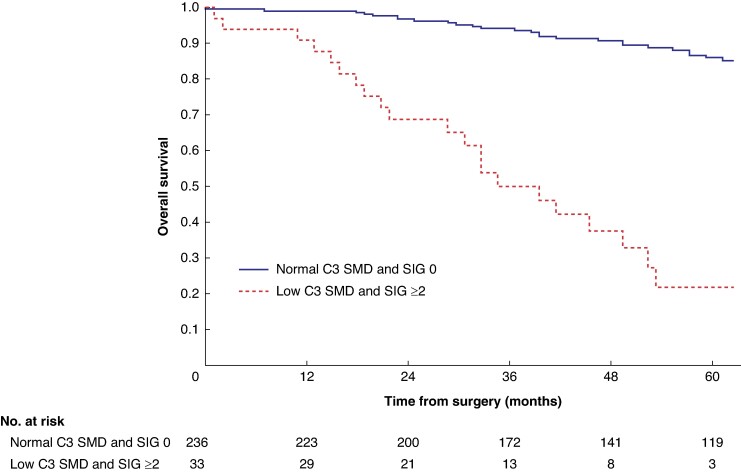
Overall survival in patients undergoing carotid endarterectomy for symptomatic carotid stenosis when subgrouped by C3 skeletal muscle density and systemic inflammatory grade (*P* < 0.001) SMD, skeletal muscle density; SIG, systemic inflammatory grade.

**Fig. 6:**
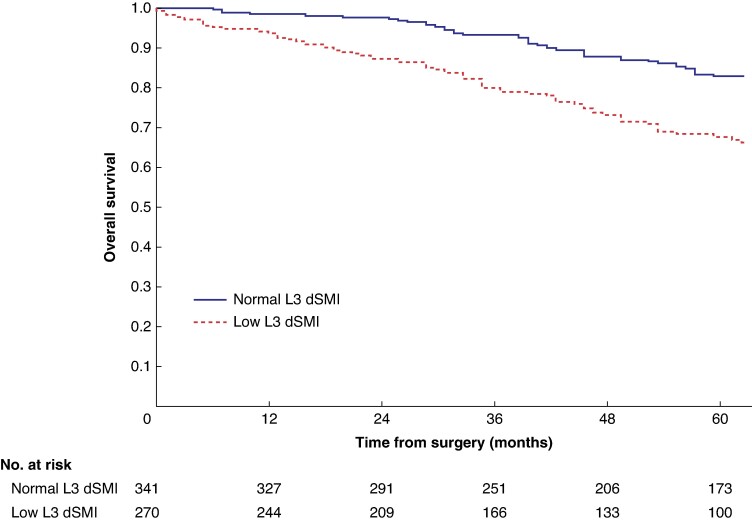
Overall survival in patients undergoing carotid endarterectomy for symptomatic carotid stenosis when subgrouped by L3 derived skeletal muscle index (*P* < 0.001) dSMI, derived skeletal muscle index.

Mean survival in the normal C3 SMI *versus* low C3 SMI subgroups was 110.3 (95% c.i. 103.3 to 117.4) *versus* 93.5 (95% c.i. 86.6 to 100.3) months (*P* = 0.002) (*Fig. 1*). Mean survival in the normal C3 SMD *versus* low C3 SMD subgroups was 109.8 (95% c.i. 103.7 to 116.0) *versus* 84.2 (95% c.i. 76.2 to 92.1) months (*P* < 0.001) (*Fig. 2*). Mean survival in the SIG 0 *versus* SIG 1 *versus* SIG greater than or equal to 2 subgroups was 110.5 (95% c.i. 104.1 to 117.0) *versus* 97.3 (95% c.i. 88.1 to 106.5) *versus* 74.9 (95% c.i. 62.7–87.1) months (*P* < 0.001) (*Fig. 3*). Patients with normal C3 SMI and SIG 0 had a mean survival of 116.3 (95% c.i. 107.9 to 124.7) months, compared with 67.8 (95% c.i. 52.4 to 82.9) months in patients with low C3 SMI and SIG greater than or equal to 2 (*P* < 0.001) (*Fig. 4*). Patients with normal C3 SMD and SIG 0 had a mean survival of 113.9 (95% c.i. 106.3 to 121.5) months, compared with 40.5 (95% c.i. 31.2 to 49.8) months in patients with low C3 SMD and SIG greater than or equal to 2 (*P* < 0.001) (*Fig. 5*). Mean survival in the normal L3 dSMI *versus* low L3 dSMI subgroups was 114.6 (95% c.i. 108.1 to 121.0) *versus* 85.3 (95% c.i. 77.9 to 106.9) months (*P* < 0.001) (*Fig. 6*).

The association between baseline clinical characteristics, CT-BC parameters, systemic inflammation, and survival is shown in *Table 4*. On multivariable analysis, age greater than or equal to 75 years (HR 2.17 (95% c.i. 1.58 to 2.97), *P* < 0.001), ASA grade greater than II (HR 2.06 (95% c.i. 1.35 to 3.12), *P* < 0.001), low C3 SMD (HR 1.84 (95% c.i. 1.33 to 2.54), *P* < 0.001), and SIG greater than or equal to 2 (HR 1.63 (95% c.i. 1.33 to 1.99), *P* < 0.001) were independently associated with increased mortality, whereas BMI greater than or equal to 25 kg/m^2^ was independently associated with decreased mortality (HR 0.64 (95% c.i. 0.47 to 0.89), *P* = 0.007).

**Table 4:** Relationship between baseline clinical characteristics, CT-derived body composition analysis parameters, systemic inflammation, and overall survival in patients undergoing carotid endarterectomy for symptomatic carotid stenosis (*n* = 618)

Covariate	*n* (%)	Univariable HR (95% c.i.)	Univariable *P*	Multivariable HR (95% c.i.)	Multivariable *P*
Age ≥75 years	188 (30.4)	2.46 (1.83,3.31)	<0.001*	2.17 (1.58,2.97)	<0.001*
Female sex	182 (29.2)	1.27 (0.93,1.73)	0.127	–	–
BMI ≥25 kg/m^2^	426 (69.4)	0.70 (0.51,0.95)	0.023*	0.64 (0.47,0.89)	<0.007*
ASA grade >II	397 (68.2)	2.36 (1.56,3.56)	<0.001*	2.06 (1.35,3.12)	<0.001*
CVA at diagnosis	306 (49.8)	1.01 (0.75,1.36)	0.947	–	–
Low C3 SMI†	328 (53.4)	1.62 (1.20,2.19)	0.002*	1.20† (0.86,1.67)	0.277
Low L3 dSMI†	270 (44.2)	2.38 (1.75,3.22)	<0.001*	1.40† (0.97,2.02)	0.071
Low C3 SMD	189 (30.7)	2.02 (1.50,2.71)	<0.001*	1.84 (1.33,2.54)	<0.001*
SIG ≥2	94 (15.3)	1.68 (1.40,2.03)	<0.001*	1.63 (1.33,1.99)	<0.001*

HRs describe hazards of all-cause mortality during the follow-up interval generated through Cox proportional hazards analysis. For covariates with more than two subgroups, the first category was considered as the reference category. *Statistically significant. **†**C3 skeletal muscle index and L3 derived skeletal muscle index were modelled separately in the multivariable model to avoid multicollinearity; values reported for the other covariates in the multivariable model pertain to the model including C3 skeletal muscle index. CT, computed tomography; CVA, cerebrovascular attack; SMI, skeletal muscle index; dSMI, derived skeletal muscle index; SMD, skeletal muscle density; SIG, systemic inflammatory grade.

Patients with normal C3 SMI and SIG 0 had 89.0% (s.e. 3.0%) %5-year survival, compared with 43.0% (s.e. 7.0%) in patients with low C3 SMI and SIG greater than or equal to 2 (*P* < 0.001). Patients with normal C3 SMD and SIG 0 had 87.0% (s.e. 2.0%) %5-year survival, compared with 26.0% (s.e. 8.0%) in patients with low C3 SMD and SIG greater than or equal to 2 (*P* < 0.001). Patients with normal L3 dSMI and SIG 0 had 89.0% (s.e. 2.0%) %5-year survival, compared with 40.0% (s.e. 7.0%) in patients with low L3 dSMI and SIG greater than or equal to 2 (*P* < 0.001).

To account for potential selection bias, patients who were excluded due to missing CT or SIG data (*n* = 352) were compared with the final study cohort (*Table S2*). There was a greater proportion of excluded patients with ASA grade greater than II (87.4% *versus* 68.3%, *P* < 0.001); however, other parameters were similar between subgroups.

## Discussion

The results of the present study describe a prognostic role for both CT-derived cervical muscle analysis and assessment of the SIR in patients undergoing carotid endarterectomy for symptomatic carotid artery disease. These novel observations in this patient cohort add to the growing body of evidence supporting the potential clinical role for CT-derived muscle analysis and assessment of the SIR in risk stratification and patient selection for major surgical intervention. It was of interest that several of the standard clinical variables (for example age and ASA grade) had larger HRs than C3 SMD and SIG. Nevertheless, these HRs were from multivariable analyses and this would indicate that the prognostic value of such standard clinical variables may be improved by inclusion of the additional variables. Additionally, by transforming cervical muscle mass to abdominal muscle mass, the present study validates the methodology used by Swartz *et al*.^23^ in a non-cancer population, which may allow for comparison with other populations where L3 CT-BC has been performed. It is likely that the inferior survival in patients with low cervical muscle mass/quality and activation of the SIR reflects increased cardiovascular morbidity, as an association between chronic inflammation and atherosclerotic disease is well established^9,26,27^.

It is proposed that presentation with TIA or CVA should be considered as an ‘end-organ’ manifestation of atherosclerosis and reflects a greater systemic burden of atherosclerotic disease. The potential clinical benefit to identifying these patients at risk of inferior cardiovascular outcome is the role of individualized secondary prevention strategies.

Identifying patients with an inferior prognosis is essential to patient selection for surgery due to the prophylactic nature of carotid intervention. To justify the exposure to peri-procedural risk, a candidate patient must be likely to have sufficient survival irrespective of intervention, to derive benefit from CVA risk reduction using surgery. The present study reports a mean survival of 40 months in patients with low C3 SMD and SIG greater than or equal to 2; in this ‘high risk’ subgroup, the benefit of intervention is mitigated by poor overall long-term survival. Identifying these higher-risk patients is likely to be of particular value in the asymptomatic carotid cohort, where the benefit of intervention is thought to be more limited than in those with symptomatic lesions^28,29^. Reproducing the results of the present study in an asymptomatic carotid cohort would be of particular interest. Furthermore, evaluation of the prognostic value of C3 SMD and SIG in a wider cohort of patients presenting with a cerebrovascular event and a likely symptomatic carotid lesion who do not undergo surgery is an important area for further research. Comparing the prognostic value of these parameters and conventional clinical assessments of fitness and long-term survival (for example age and ASA grade) would allow for their clinical utility in patient selection to be assessed more accurately.

The prognostic value of CT-BC and the SIR in similar patient cohorts has been previously described; two studies report inferior outcomes relating to sarcopenia as defined by masseter area^16,17^ and two studies report inferior outcomes in patients with an elevated preoperative NLR^18,19^. The present study is the first to report the combined prognostic value of both of these factors, which have been extensively reported in patients with cancer and previously reported in patients with abdominal aortic aneurysms and with peripheral arterial disease^14,30^. The predominant cervical CT-BC parameter reported in patients with head and neck cancer is the C3 SMI rather than masseter area and is recommended by the authors of a recent meta-analysis^15^. Normalization of muscle areas is well established in the measurement of CT-BC parameters; therefore, the present study employs more robust methodology than previous studies. Similarly, whilst the use of NLR and mGPS as an assessment of the SIR from myeloid and liver tissue respectively has been widely reported, SIG incorporating both represents a more comprehensive assessment.

The prevalence, associations, and prognostic value of sarcopenia have been assessed in a range of disease states. The majority of studies report on patients with cancer, where standardized thresholds of skeletal muscle mass and density at L3 have been proposed and validated^24^. The present study analyses muscle mass and density at C3, as abdominal CT images were not available for patients undergoing assessment for carotid endarterectomy. Nonetheless, sarcopenia is a systemic disease process, which in principle should affect skeletal muscle globally, thereby supporting the rationale for measuring cervical musculature. Furthermore, multiple studies in patients with head and neck cancer report outcomes based on the C3 SMI^15^. The prognostic value of the L3 dSMI using a previously proposed method of derivation was also reported^23^. There appear to be shared risk factors that contribute to similar prevalences of sarcopenia irrespective of disease state^31^. By reporting and validating the derivation of L3 dSMI from C3 SMI, the results of the present study enable the potential comparison of muscle mass between cohorts using different measurement techniques, as well as contributing to the evidence base that sarcopenia can be assessed at a variety of vertebral levels, depending on the availability of relevant imaging.

The present study is limited by its retrospective design, the relatively small sample size in certain subgroups, and data-derived thresholds for CT-BC parameters. The use of arterial-phase images may limit comparison with external cohorts, due to the potential confounding effect of CT phase on SMD measurement^32^. Patients with asymptomatic carotid lesions were not included, thereby limiting the application of conclusions to the entire population of patients undergoing carotid endarterectomy; however, the cohort reflects contemporary real-world UK practice^2^. Lack of a ‘healthy’ control group potentially limits the validity of the conclusions; however, the exposure to ionizing radiation required for CT makes this non-feasible. Data detailing specific cause of death were not available; therefore, it is inferred that the likely increased mortality is cardiovascular in aetiology based on previous literature (however, this is a limitation and potential source of bias). The use of ASA grade to measure co-morbidity is a potential source of bias in a retrospective study due to variable implementation of the ASA grade, which is particularly apparent in the context of patients who have had recent cerebrovascular events, given that fewer patients than expected had an ASA grade greater than II.

Cervical CT-derived muscle mass and density, and readily available markers of systemic inflammation, such as SIG, may be used to identify patients with an inferior long-term prognosis after carotid endarterectomy. These factors may offer clinical utility in selecting patients likely to benefit from surgery and in developing novel secondary prevention strategies, though large prospective validation of the present results is required.

## Supplementary Material

zrae114_Supplementary_Data

## Data Availability

Data could be made available on request to the corresponding author.

## References

[1] Naylor R, Rantner B, Ancetti S, de Borst GJ, De Carlo M, Halliday A et al Editor’s Choice—European Society for Vascular Surgery (ESVS) 2023 clinical practice guidelines on the management of atherosclerotic carotid and vertebral artery disease. Eur J Vasc Endovasc Surg 2023;65:7–111 35598721 10.1016/j.ejvs.2022.04.011

[2] Waton S, Johal A, Birmpili P, Li Q, Atkins E, Cromwell DA et al *National Vascular Registry: 2022 Annual Report*. 2022. https://www.vsqip.org.uk/wp-content/uploads/2024/04/NVR-2022-Annual-Report.pdf#:~:text=NVR%202022%20Annual%20Report.%20National%20Vascular%20Registry%20Clinical (accessed 2 March 2024)

[3] Cruz-Jentoft AJ, Bahat G, Bauer J, Boirie Y, Bruyère O, Cederholm T et al Sarcopenia: revised European consensus on definition and diagnosis. Age Ageing 2019;48:16–31 30312372 10.1093/ageing/afy169 PMC6322506

[4] Heard RSM, Ramsay G, Hildebrand DR. Sarcopaenia in surgical populations: a review. Surgeon 2017;15:366–371 28684167 10.1016/j.surge.2017.06.001

[5] Heard R, Black D, Ramsay G, Scott N, Hildebrand D. The prevalence of sarcopaenia in a vascular surgical patient cohort and its impact on outcome. Surgeon 2018;16:325–332 29669697 10.1016/j.surge.2018.03.001

[6] Abbass T, Dolan RD, McMillan DC. Computed tomography-derived body composition analysis in patients with advanced cancer: clinical utility and future research. Curr Opin Support Palliat Care 2020;14:309–315 33105241 10.1097/SPC.0000000000000529

[7] Bradley NA, Roxburgh CSD, McMillan DC, Guthrie GJK. The relationship between pre-operative psoas and skeletal muscle parameters and survival following endovascular aneurysm repair: a systematic review and meta-analysis. Sci Reports 2022;12:16663 10.1038/s41598-022-20490-3 PMC9534993 36198699

[8] Bradley NA, Walter A, Dolan R, Wilson A, Siddiqui T, Roxburgh CSD et al Evaluation of the prognostic value of computed tomography-derived body composition in patients undergoing endovascular aneurysm repair. J Cachexia Sarcopenia Muscle 2023;14:1836–1847 37221439 10.1002/jcsm.13262 PMC10401537

[9] Libby P, Ridker PM, Maseri A. Inflammation and atherosclerosis. Circulation 2002;105:1135–1143 11877368 10.1161/hc0902.104353

[10] Guthrie GJK, Charles KA, Roxburgh CSD, Horgan PG, McMillan DC, Clarke SJ. The systemic inflammation-based neutrophil-lymphocyte ratio: experience in patients with cancer. Crit Rev Oncol Hematol 2013;88:218–230 23602134 10.1016/j.critrevonc.2013.03.010

[11] Proctor MJ, Morrison DS, Talwar D, Balmer SM, O'Reilly DSJ, Foulis AK et al An inflammation-based prognostic score (mGPS) predicts cancer survival independent of tumour site: a Glasgow Inflammation Outcome Study. Br J Cancer 2011;104:726–734 21266974 10.1038/sj.bjc.6606087 PMC3049591

[12] Bradley NA, Walter A, Wilson A, Wilson A, Siddiqui T, Roxburgh CSD et al The prognostic value of preoperative systemic inflammation-based scoring in patients undergoing endovascular repair of abdominal aortic aneurysm. J Vasc Surg 2023;78:362–369.e2 37086821 10.1016/j.jvs.2023.04.018

[13] Dolan RD, Almasaudi AS, Dieu LB, Horgan PG, McSorley ST, McMillan DC. The relationship between computed tomography-derived body composition, systemic inflammatory response, and survival in patients undergoing surgery for colorectal cancer. J Cachexia Sarcopenia Muscle 2019;10:111–122 30460764 10.1002/jcsm.12357 PMC6438413

[14] Bradley NA, Walter A, Wilson A, Siddiqui T, Roxburgh CSD, McMillan DC et al The relationship between CT-derived body composition, systemic inflammation, and survival in patients with abdominal aortic aneurysm. J Vasc Surg 2023;78:937–944.e4 37385355 10.1016/j.jvs.2023.06.012

[15] Takenaka Y, Takemoto N, Oya R, Inohara H. Prognostic impact of sarcopenia in patients with head and neck cancer treated with surgery or radiation: a meta-analysis. PLoS One 2021;16:e0259288 34714876 10.1371/journal.pone.0259288 PMC8555817

[16] Oksala NKJ, Lindström I, Khan N, Pihlajaniemi VJ, Lyytikäinen L-P, Pienimäki J-P et al Pre-operative masseter area is an independent predictor of long-term survival after carotid endarterectomy. Eur J Vasc Endovasc Surg 2019;57:331–338 30583960 10.1016/j.ejvs.2018.11.011

[17] Hogenbirk RNM, Banning LBD, Visser A, Jager-Wittenaar H, Pol RA, Zeebregts CJ et al Association between masseter muscle area and thickness and outcome after carotid endarterectomy: a retrospective cohort study. J Clin Med 2022;11:3087 35683474 10.3390/jcm11113087 PMC9181694

[18] King AH, Kim AH, Kwan S, Lee J, Schmaier AH, Kumins NH et al Elevated neutrophil to lymphocyte ratio is associated with worse outcomes after carotid endarterectomy in asymptomatic patients. J Stroke Cerebrovasc Dis 2021;30:106120 34597986 10.1016/j.jstrokecerebrovasdis.2021.106120

[19] Casanova N, Diaz-Duran C, Nieto L, Llort C, Elosua R, Clara A et al Predictive value of complete blood count-derived inflammatory markers for 5-year survival after carotid endarterectomy: implications for practice. Angiology 2022;73:675–681 35089092 10.1177/00033197211067581

[20] Hackett NJ, De Oliveira GS, Jain UK, Kim JYS. ASA class is a reliable independent predictor of medical complications and mortality following surgery. Int J Surg 2015;18:184–190 25937154 10.1016/j.ijsu.2015.04.079

[21] van Rijn-Dekker MI, van den Bosch L, van den Hoek JGM, Bijl HP, van Aken ESM, van der Hoorn A et al Impact of sarcopenia on survival and late toxicity in head and neck cancer patients treated with radiotherapy. Radiother Oncol 2020;147:103–110 32251949 10.1016/j.radonc.2020.03.014

[22] Schneider CA, Rasband WS, Eliceiri KW. NIH image to ImageJ: 25 years of image analysis. Nat Methods 2012;9:671–675 22930834 10.1038/nmeth.2089 PMC5554542

[23] Swartz JE, Pothen AJ, Wegner I, Smid EJ, Swart KMA, de Bree R et al Feasibility of using head and neck CT imaging to assess skeletal muscle mass in head and neck cancer patients. Oral Oncol 2016;62:28–33 27865369 10.1016/j.oraloncology.2016.09.006

[24] Martin L, Birdsell L, MacDonald N, Reiman T, Clandinin MT, McCargar LJ et al Cancer cachexia in the age of obesity: skeletal muscle depletion is a powerful prognostic factor, independent of body mass index. J Clin Oncol 2013;31:1539–1547 23530101 10.1200/JCO.2012.45.2722

[25] Golder AM, McMillan DC, Park JH, Mansouri D, Horgan PG, Roxburgh CS. The prognostic value of combined measures of the systemic inflammatory response in patients with colon cancer: an analysis of 1700 patients. Br J Cancer 2021;124:1828–1835 33762720 10.1038/s41416-021-01308-x PMC8144393

[26] Fatkhullina AR, Peshkova IO, Koltsova EK. The role of cytokines in the development of atherosclerosis. Biochemistry (Mosc) 2016;81:1358–1370 27914461 10.1134/S0006297916110134 PMC5471837

[27] Falk E . Pathogenesis of atherosclerosis. J Am Coll Cardiol 2006;47:C7–C12 16631513 10.1016/j.jacc.2005.09.068

[28] Halliday A, Bulbulia R, Bonati LH, Chester J, Cradduck-Bamford A, Peto R et al Second asymptomatic carotid surgery trial (ACST-2): a randomised comparison of carotid artery stenting versus carotid endarterectomy. Lancet 2021;398:1065–1073 34469763 10.1016/S0140-6736(21)01910-3 PMC8473558

[29] Halliday A, Harrison M, Hayter E, Kong X, Mansfield A, Marro J et al 10-year stroke prevention after successful carotid endarterectomy for asymptomatic stenosis (ACST-1): a multicentre randomised trial. Lancet 2010;376:1074–1084 20870099 10.1016/S0140-6736(10)61197-X PMC2956884

[30] Bradley NA, Walter A, Roxburgh CSD, McMillan DC, Guthrie GJK. The relationship between clinical frailty score, CT-derived body composition, systemic inflammation, and survival in patients with chronic limb threatening ischaemia. Ann Vasc Surg 2023;104:18–26 37356659 10.1016/j.avsg.2023.06.012

[31] Bradley NA, McGovern J, Dolan RD, Golder AM, Roxburgh CSD, Guthrie GJK et al CT-derived body composition: differential association with disease, age and inflammation in a retrospective cohort study. PLoS One 2024;19:e0300038 38512880 10.1371/journal.pone.0300038 PMC10956827

[32] Rollins KE, Javanmard-Emamghissi H, Awwad A, Macdonald IA, Fearon KCH, Lobo DN. Body composition measurement using computed tomography: does the phase of the scan matter? Nutrition 2017;41:37–44 28760426 10.1016/j.nut.2017.02.011

