# Supplementary material for: Relationship between CT-derived cervical muscle mass and quality, systemic inflammation, and survival in symptomatic patients undergoing carotid endarterectomy
Source: BJS Open. 2024 Oct 23;8(5):zrae114. doi: 10.1093/bjsopen/zrae114 (PMC11498072; doi:10.1093/bjsopen/zrae114)
Supplement: zrae114_Supplementary_Data [file zrae114_supplementary_data.docx]

**The relationship between CT-derived cervical muscle mass and quality, systemic inflammation, and survival in symptomatic patients undergoing carotid endarterectomy.**

N A Bradley^1^, K Dosanj^2^, S Yen Ming Chan^3^, A Wilson^4^, T Siddiqui^5^, R Forsythe^6^, C S D Roxburgh^7^, D C McMillan^8^, G J K Guthrie^9^.

1. Clinical Research Fellow, University of Glasgow
2. Academic Foundation Doctor, NHS Tayside
3. Vascular Specialty Registrar, NHS Lothian
4. Consultant Vascular Surgeon, NHS Grampian
5. Consultant Vascular Surgeon, NHS Lanarkshire
6. Consultant Vascular Surgeon, NHS Lothian
7. Professor of Surgery, University of Glasgow
8. Professor of Surgical Science, University of Glasgow
9. Consultant Vascular Surgeon, NHS Tayside, Honorary Clinical Senior Lecturer, University of Glasgow

Corresponding Author : Nicholas Bradley, Room 2.56, New Lister Building, Glasgow Royal Infirmary, Glasgow, G4 0SF. [Nicholasandrew.bradley@glasgow.ac.uk](mailto:Nicholasandrew.bradley@glasgow.ac.uk)

**Supplementary Materials - Index**

| **Supplementary Figures and Tables** |  |
| --- | --- |
| Table S1 | *page 2* |
| Table S2 | *page 3* |

**Supplementary Figures and Tables**

| **Table S1:** The calculation of inflammation-based prognostic scores using pre-operative blood results. | | |
| --- | --- | --- |
| Neutrophil:Lymphocyte Ratio (NLR) | Absolute Neutrophil Count / Absolute Lymphocyte Count | |
|  | | |
| Modified Glasgow Prognostic Score (mGPS) | mGPS 0 | CRP ≤ 10mg/L |
|  | mGPS 1 | CRP > 10mg/L and Albumin > 35 g/L |
|  | mGPS 2 | CRP > 10mg/L and Albumin < 35 g/L |
|  | | |
| Systemic Inflammatory Grade (SIG) | SIG 0 | mGPS 0 and NLR < 3 |
|  | SIG 1 | mGPS 0 and NLR 3 – 5  or  mGPS 1 and NLR < 3 |
|  | SIG 2 | mGPS 0 and NLR > 5  or  mGPS 2 and NLR < 3  or  mGPS 1 and NLR 3 - 5 |
|  | SIG 3 | mGPS 1 and NLR > 5  or  mGPS 2 and NLR 3 - 5 |
|  | SIG 4 | mGPS 2 and NLR > 5 |

**Table S2:** The comparison between the final study cohort and patients excluded from the study on the basis of missing CT-BC parameters or SIG.

|  | **Excluded Patients**  **(n = 352)** | **Final Study Cohort**  **(n = 618)** | ***p*** |
| --- | --- | --- | --- |
| **Age**  < 75  ≥ 75 | 239 (68%)  111 (32%) | 430 (70%)  188 (30% | 0.68 |
| **Sex**  Male  Female | 249 (71%)  103 (29%) | 438 (71%)  180 (29%) | 0.97 |
| **BMI**  < 25 kg/m^2^  ≥ 25 kg/m^2^ | 76 (25%)  231 (75%) | 187 (31%)  426 (69%) | 0.07 |
| **ASA**  ≤ 2  > 2 | 37 (13%)  260 (87%) | 184 (32%)  397 (68%) | **<0.001** |
| **Indication**  CVA  TIA | 45 (43%)  59 (57%) | 219 (48%)  238 (52%) | 0.11 |
| *p* values generated through linear-by-linear Chi Squared analyses comparing proportion of each covariate within each subgroup. BMI; Body Mass Index. ASA; American society of Anaesthesiologists score. CVA; cerebrovascular attack. TIA; transient ischaemic attack. | | | |
